# 2,4-Bis(4-propoxyphen­yl)-3-aza­bicyclo­[3.3.1]nonan-9-one

**DOI:** 10.1107/S1600536811007483

**Published:** 2011-03-05

**Authors:** P. Parthiban, V. Ramkumar, Yeon Tae Jeong

**Affiliations:** a Department of Image Science and Engineering, Pukyong National University, Busan 608 739, Republic of Korea; b Department of Chemistry, IIT Madras, Chennai, TamilNadu, India

## Abstract

In the title compound, C_26_H_33_NO_3_, a crystallographic mirror plane bis­ects the mol­ecule (two C atoms, one O atom and one N atom lie on the mirror plane). The mol­ecule exists in a twin-chair conformation with equatorial orientations of the 4-propoxyphenyl groups. The dihedral angle between the 4-propoxyphenyl groups is 31.58 (3)°.

## Related literature

For background to 3-aza­bicyclo­nona­nes, see: Jeyaraman & Avila (1981 ▶); Barker *et al.* (2005 ▶); Parthiban *et al.* (2009*a* ▶, 2010*b* ▶,*c* ▶). For related stuctures, see: Parthiban *et al.* (2009*b* ▶,*c* ▶, 2010*a* ▶); Smith-Verdier *et al.* (1983 ▶); Padegimas & Kovacic (1972 ▶). For ring puckering and asymmetry parameters, see: Cremer & Pople (1975 ▶); Nardelli (1983 ▶).
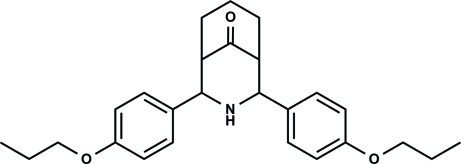


## Experimental

### 

#### Crystal data

C_26_H_33_NO_3_
*M* *_r_* = 407.53
Orthorhombic, 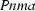

*a* = 7.3846 (4) Å
*b* = 29.3963 (19) Å
*c* = 10.2739 (7) Å
*V* = 2230.3 (2) Å^3^
*Z* = 4
Mo *K*α radiation
μ = 0.08 mm^−1^
*T* = 298 K
0.25 × 0.22 × 0.20 mm

#### Data collection

Bruker APEXII CCD diffractometer
Absorption correction: multi-scan (*SADABS*; Bruker, 2004 ▶) *T*_min_ = 0.981, *T*_max_ = 0.985
7260 measured reflections
1860 independent reflections
1121 reflections with *I* > 2σ(*I*)
*R*_int_ = 0.047

#### Refinement

*R*[*F* ^2^ > 2σ(*F* ^2^)] = 0.044
*wR*(*F* ^2^) = 0.104
*S* = 1.00
1860 reflections
146 parameters
H atoms treated by a mixture of independent and constrained refinement
Δρ_max_ = 0.14 e Å^−3^
Δρ_min_ = −0.21 e Å^−3^

### 

Data collection: *APEX2* (Bruker, 2004 ▶); cell refinement: *SAINT-Plus* (Bruker, 2004 ▶); data reduction: *SAINT-Plus* and *XPREP* (Bruker, 2004 ▶); program(s) used to solve structure: *SHELXS97* (Sheldrick, 2008 ▶); program(s) used to refine structure: *SHELXL97* (Sheldrick, 2008 ▶); molecular graphics: *ORTEP-3* (Farrugia, 1997 ▶); software used to prepare material for publication: *SHELXL97*.

## Supplementary Material

Crystal structure: contains datablocks global, I. DOI: 10.1107/S1600536811007483/hb5803sup1.cif

Structure factors: contains datablocks I. DOI: 10.1107/S1600536811007483/hb5803Isup2.hkl

Additional supplementary materials:  crystallographic information; 3D view; checkCIF report

## Figures and Tables

**Fig. 1.**
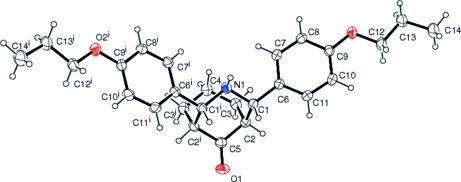
: Anistropic displacement representation of the molecule with atoms represented with 30% probability ellipsoids. Symmetry code: (i) x, 1/2–y, z.

## supplementary crystallographic information

### Comment

3-Azabicyclononanes are important class of heterocycles due to their broad spectrum of biological activities such as antibacterial, antimycobacterial, antifungal, anticancer, antitussive, anti-inflammatory, sedative, antipyretic and calcium antagonistic activities (Jeyaraman & Avila, 1981; Barker *et al.*, 2005; Parthiban *et al.*, 2009*a*, 2010*b*,*c*). Since the stereochemistry plays an important role in biological actions, it is important to establish the stereochemistry of the synthesized biologically potent molecules. Owing to the diverse possibilities in conformation of the 3-azabicycles, *viz*., chair-chair (Parthiban *et al.*, 2009*a*), chair-boat (Smith-Verdier *et al.*, 1983) and boat-boat (Padegimas & Kovacic, 1972). This crystal study has been carried out to expose the conformation of the title bicyclic compound.

The analysis of torsion angles, asymmetry parameters and puckering parameters calculated for the title compound shows that the piperidine ring adopts near ideal chair conformation with a total puckering amplitude, Q_T_ of 0.615 (2)Å and the phase angle θ is 0.00 (1)°. (Cremer & Pople, 1975). The smallest displacement asymmetry parameters q_2_ and q_3_ are 0.00 and 0.615 (2)°, respectively (Nardelli, 1983). However, the cyclohexane ring deviates from the ideal chair conformation according to Cremer and Pople by Q_T_ = 0.562 (2) and θ = 16.8 (2)° (Cremer & Pople, 1975) as well as Nardelli by q_2_ = 0.162 (2) and q_3_ = 0.538 (2)° (Nardelli, 1983). Hence, the title compound C_28_ H_33_ N O_3_, exists in a twin-chair conformation with equatorial orientation of 4-propoxyphenyl groups on the heterocycle and are orientated at an angle of 31.58 (3)° to each other. The torsion angle of C3—C2—C1—C6 are 64.09 (3)°. The crystal crystal packing is stabilized by weak van der Waals interaction.

### Experimental

To the warm solution of 0.075 mol (5.78 g) ammonium acetate in 50 ml of absolute ethanol, 0.1 mol (16.42 g/15.80 ml)of *para*-n-propoxybenzaldehyde and 0.05 mol (4.90 g/5.18 ml) of cyclohexanone were added. The mixture was gently warmed on a hot plate at 303–308 K (30–35° C) with moderate stirring till the complete consumption of the starting materials, monitored by TLC. At the end, the crude azabicyclic ketone was separated by filtration and washed with 1:5 cold ethanol-ether mixture. Colourless blocks of the title compound were obtained by recrystallization from ethanol.

### Refinement

Nitrogen H atoms were located in a difference Fourier map and refined isotropically. Other hydrogen atoms were fixed geometrically and allowed to ride on the parent carbon atoms,with aromatic C—H =0.93 Å, aliphatic C—H = 0.98Å and methylene C—H = 0.97 Å. The displacement parameters were set for phenyl,methylene and aliphatic H atoms at *U*_iso_(H) = 1.2*U*_eq_(C).

### Crystal data

**Table:**

C_26_H_33_NO_3_	*F*(000) = 880
*M**_r_* = 407.53	*D*_x_ = 1.214 Mg m^−^^3^
Orthorhombic, *P**n**m**a*	Mo *K*α radiation, λ = 0.71073 Å
Hall symbol: -P 2ac 2n	Cell parameters from 1042 reflections
*a* = 7.3846 (4) Å	θ = 2.3–20.8°
*b* = 29.3963 (19) Å	µ = 0.08 mm^−^^1^
*c* = 10.2739 (7) Å	*T* = 298 K
*V* = 2230.3 (2) Å^3^	Block, colourless
*Z* = 4	0.25 × 0.22 × 0.20 mm

### Data collection

**Table:**

Bruker APEXII CCD diffractometer	1860 independent reflections
Radiation source: fine-focus sealed tube	1121 reflections with *I* > 2σ(*I*)
graphite	*R*_int_ = 0.047
φ and ω scans	θ_max_ = 26.2°, θ_min_ = 2.1°
Absorption correction: multi-scan (*SADABS*; Bruker, 2004)	*h* = −8→8
*T*_min_ = 0.981, *T*_max_ = 0.985	*k* = −31→32
7260 measured reflections	*l* = −12→10

### Refinement

**Table:**

Refinement on *F*^2^	Primary atom site location: structure-invariant direct methods
Least-squares matrix: full	Secondary atom site location: difference Fourier map
*R*[*F*^2^ > 2σ(*F*^2^)] = 0.044	Hydrogen site location: inferred from neighbouring sites
*wR*(*F*^2^) = 0.104	H atoms treated by a mixture of independent and constrained refinement
*S* = 1.00	*w* = 1/[σ^2^(*F*_o_^2^) + (0.0404*P*)^2^ + 0.4368*P*] where *P* = (*F*_o_^2^ + 2*F*_c_^2^)/3
1860 reflections	(Δ/σ)_max_ < 0.001
146 parameters	Δρ_max_ = 0.14 e Å^−^^3^
0 restraints	Δρ_min_ = −0.20 e Å^−^^3^

### Special details

**Table:**

Geometry. All e.s.d.'s (except the e.s.d. in the dihedral angle between two l.s. planes) are estimated using the full covariance matrix. The cell e.s.d.'s are taken into account individually in the estimation of e.s.d.'s in distances, angles and torsion angles; correlations between e.s.d.'s in cell parameters are only used when they are defined by crystal symmetry. An approximate (isotropic) treatment of cell e.s.d.'s is used for estimating e.s.d.'s involving l.s. planes.
Refinement. Refinement of *F*^2^ against ALL reflections. The weighted *R*-factor *wR* and goodness of fit *S* are based on *F*^2^, conventional *R*-factors *R* are based on *F*, with *F* set to zero for negative *F*^2^. The threshold expression of *F*^2^ > σ(*F*^2^) is used only for calculating *R*-factors(gt) *etc*. and is not relevant to the choice of reflections for refinement. *R*-factors based on *F*^2^ are statistically about twice as large as those based on *F*, and *R*- factors based on ALL data will be even larger.

### Fractional atomic coordinates and isotropic or equivalent isotropic displacement parameters (Å^2^)

**Table:**

	*x*	*y*	*z*	*U*_iso_*/*U*_eq_	
C1	0.6512 (3)	0.29134 (6)	0.6070 (2)	0.0384 (5)	
H1	0.6934	0.2893	0.6972	0.046*	
C2	0.8207 (3)	0.29225 (7)	0.5180 (2)	0.0426 (6)	
H2	0.8944	0.3188	0.5408	0.051*	
C3	0.7815 (3)	0.29351 (7)	0.3708 (2)	0.0474 (6)	
H3A	0.7004	0.3187	0.3529	0.057*	
H3B	0.8940	0.2993	0.3249	0.057*	
C4	0.6973 (4)	0.2500	0.3174 (3)	0.0482 (8)	
H4A	0.7093	0.2500	0.2234	0.058*	
H4B	0.5691	0.2500	0.3377	0.058*	
C5	0.9286 (4)	0.2500	0.5451 (3)	0.0437 (8)	
C6	0.5396 (3)	0.33407 (7)	0.5949 (2)	0.0364 (5)	
C7	0.3900 (3)	0.33809 (7)	0.5140 (2)	0.0418 (6)	
H7	0.3506	0.3130	0.4666	0.050*	
C8	0.2990 (3)	0.37892 (7)	0.5032 (2)	0.0426 (6)	
H8	0.1988	0.3811	0.4488	0.051*	
C9	0.3553 (3)	0.41666 (7)	0.5724 (2)	0.0389 (6)	
C10	0.5009 (3)	0.41290 (7)	0.6550 (2)	0.0480 (6)	
H10	0.5384	0.4378	0.7040	0.058*	
C11	0.5911 (3)	0.37188 (7)	0.6647 (2)	0.0462 (6)	
H11	0.6901	0.3697	0.7204	0.055*	
C12	0.3046 (3)	0.49521 (7)	0.6265 (2)	0.0484 (6)	
H12A	0.4284	0.5039	0.6070	0.058*	
H12B	0.2959	0.4889	0.7190	0.058*	
C13	0.1778 (3)	0.53290 (7)	0.5904 (2)	0.0578 (7)	
H13A	0.0545	0.5237	0.6096	0.069*	
H13B	0.1863	0.5386	0.4976	0.069*	
C14	0.2201 (4)	0.57630 (8)	0.6634 (3)	0.0768 (9)	
H14A	0.2116	0.5708	0.7554	0.115*	
H14B	0.1350	0.5995	0.6390	0.115*	
H14C	0.3405	0.5861	0.6423	0.115*	
N1	0.5477 (4)	0.2500	0.5790 (3)	0.0405 (7)	
O1	1.0831 (3)	0.2500	0.5851 (2)	0.0633 (7)	
O2	0.25630 (19)	0.45579 (5)	0.55359 (16)	0.0531 (5)	
H1N	0.458 (4)	0.2500	0.623 (3)	0.029 (9)*	

### Atomic displacement parameters (Å^2^)

**Table:**

	*U*^11^	*U*^22^	*U*^33^	*U*^12^	*U*^13^	*U*^23^
C1	0.0415 (12)	0.0358 (12)	0.0380 (13)	−0.0030 (10)	−0.0027 (10)	−0.0016 (10)
C2	0.0377 (13)	0.0352 (12)	0.0548 (16)	−0.0049 (10)	0.0003 (11)	−0.0056 (11)
C3	0.0497 (14)	0.0433 (13)	0.0492 (15)	0.0036 (11)	0.0091 (12)	0.0038 (12)
C4	0.055 (2)	0.049 (2)	0.0407 (19)	0.000	0.0047 (16)	0.000
C5	0.0354 (19)	0.049 (2)	0.047 (2)	0.000	0.0018 (16)	0.000
C6	0.0379 (12)	0.0346 (12)	0.0368 (13)	−0.0022 (10)	0.0005 (10)	−0.0019 (10)
C7	0.0445 (13)	0.0362 (13)	0.0445 (14)	−0.0045 (11)	−0.0023 (11)	−0.0051 (11)
C8	0.0439 (13)	0.0424 (14)	0.0415 (14)	−0.0020 (11)	−0.0081 (11)	−0.0027 (11)
C9	0.0413 (13)	0.0318 (13)	0.0436 (14)	−0.0017 (10)	0.0042 (11)	0.0011 (10)
C10	0.0508 (15)	0.0388 (14)	0.0543 (16)	−0.0033 (11)	−0.0069 (13)	−0.0129 (12)
C11	0.0435 (14)	0.0436 (14)	0.0515 (15)	0.0004 (11)	−0.0117 (12)	−0.0081 (12)
C12	0.0519 (14)	0.0357 (13)	0.0575 (15)	−0.0046 (11)	0.0027 (12)	−0.0054 (12)
C13	0.0594 (15)	0.0400 (14)	0.0740 (18)	0.0013 (12)	0.0019 (14)	−0.0047 (13)
C14	0.0743 (19)	0.0471 (15)	0.109 (3)	0.0032 (14)	−0.0007 (17)	−0.0183 (16)
N1	0.0371 (16)	0.0360 (16)	0.0483 (18)	0.000	0.0066 (15)	0.000
O1	0.0369 (14)	0.0686 (16)	0.0845 (18)	0.000	−0.0115 (13)	0.000
O2	0.0604 (10)	0.0345 (9)	0.0644 (11)	0.0053 (7)	−0.0124 (8)	−0.0074 (8)

### Geometric parameters (Å, °)

**Table:**

C1—N1	1.464 (2)	C8—C9	1.381 (3)
C1—C6	1.507 (3)	C8—H8	0.9300
C1—C2	1.551 (3)	C9—C10	1.375 (3)
C1—H1	0.9800	C9—O2	1.376 (2)
C2—C5	1.502 (3)	C10—C11	1.381 (3)
C2—C3	1.540 (3)	C10—H10	0.9300
C2—H2	0.9800	C11—H11	0.9300
C3—C4	1.524 (3)	C12—O2	1.425 (2)
C3—H3A	0.9700	C12—C13	1.497 (3)
C3—H3B	0.9700	C12—H12A	0.9700
C4—C3^i^	1.524 (3)	C12—H12B	0.9700
C4—H4A	0.9700	C13—C14	1.512 (3)
C4—H4B	0.9700	C13—H13A	0.9700
C5—O1	1.213 (3)	C13—H13B	0.9700
C5—C2^i^	1.502 (3)	C14—H14A	0.9600
C6—C11	1.377 (3)	C14—H14B	0.9600
C6—C7	1.387 (3)	C14—H14C	0.9600
C7—C8	1.380 (3)	N1—C1^i^	1.464 (2)
C7—H7	0.9300	N1—H1N	0.80 (3)
			
N1—C1—C6	112.97 (17)	C7—C8—H8	119.6
N1—C1—C2	108.69 (19)	C9—C8—H8	119.6
C6—C1—C2	112.25 (17)	C10—C9—O2	124.69 (19)
N1—C1—H1	107.6	C10—C9—C8	119.24 (19)
C6—C1—H1	107.6	O2—C9—C8	116.06 (19)
C2—C1—H1	107.6	C9—C10—C11	119.5 (2)
C5—C2—C3	107.6 (2)	C9—C10—H10	120.3
C5—C2—C1	107.73 (19)	C11—C10—H10	120.3
C3—C2—C1	115.30 (17)	C6—C11—C10	122.3 (2)
C5—C2—H2	108.7	C6—C11—H11	118.9
C3—C2—H2	108.7	C10—C11—H11	118.9
C1—C2—H2	108.7	O2—C12—C13	108.35 (18)
C4—C3—C2	114.2 (2)	O2—C12—H12A	110.0
C4—C3—H3A	108.7	C13—C12—H12A	110.0
C2—C3—H3A	108.7	O2—C12—H12B	110.0
C4—C3—H3B	108.7	C13—C12—H12B	110.0
C2—C3—H3B	108.7	H12A—C12—H12B	108.4
H3A—C3—H3B	107.6	C12—C13—C14	111.9 (2)
C3—C4—C3^i^	114.1 (3)	C12—C13—H13A	109.2
C3—C4—H4A	108.7	C14—C13—H13A	109.2
C3^i^—C4—H4A	108.7	C12—C13—H13B	109.2
C3—C4—H4B	108.7	C14—C13—H13B	109.2
C3^i^—C4—H4B	108.7	H13A—C13—H13B	107.9
H4A—C4—H4B	107.6	C13—C14—H14A	109.5
O1—C5—C2^i^	124.19 (13)	C13—C14—H14B	109.5
O1—C5—C2	124.19 (13)	H14A—C14—H14B	109.5
C2^i^—C5—C2	111.6 (3)	C13—C14—H14C	109.5
C11—C6—C7	117.62 (19)	H14A—C14—H14C	109.5
C11—C6—C1	118.61 (18)	H14B—C14—H14C	109.5
C7—C6—C1	123.75 (19)	C1—N1—C1^i^	112.2 (2)
C8—C7—C6	120.7 (2)	C1—N1—H1N	108.6 (10)
C8—C7—H7	119.7	C1^i^—N1—H1N	108.6 (10)
C6—C7—H7	119.7	C9—O2—C12	118.21 (16)
C7—C8—C9	120.7 (2)		
			
N1—C1—C2—C5	−58.5 (2)	C1—C6—C7—C8	−177.0 (2)
C6—C1—C2—C5	175.77 (19)	C6—C7—C8—C9	0.2 (3)
N1—C1—C2—C3	61.6 (2)	C7—C8—C9—C10	−1.6 (3)
C6—C1—C2—C3	−64.1 (2)	C7—C8—C9—O2	179.35 (19)
C5—C2—C3—C4	52.0 (3)	O2—C9—C10—C11	−179.3 (2)
C1—C2—C3—C4	−68.2 (3)	C8—C9—C10—C11	1.7 (3)
C2—C3—C4—C3^i^	−42.1 (3)	C7—C6—C11—C10	−0.9 (3)
C3—C2—C5—O1	115.5 (3)	C1—C6—C11—C10	177.2 (2)
C1—C2—C5—O1	−119.6 (3)	C9—C10—C11—C6	−0.5 (3)
C3—C2—C5—C2^i^	−65.1 (3)	O2—C12—C13—C14	−179.8 (2)
C1—C2—C5—C2^i^	59.8 (3)	C6—C1—N1—C1^i^	−172.82 (15)
N1—C1—C6—C11	154.5 (2)	C2—C1—N1—C1^i^	61.9 (3)
C2—C1—C6—C11	−82.1 (2)	C10—C9—O2—C12	−1.4 (3)
N1—C1—C6—C7	−27.5 (3)	C8—C9—O2—C12	177.64 (18)
C2—C1—C6—C7	95.8 (2)	C13—C12—O2—C9	−179.52 (18)
C11—C6—C7—C8	1.0 (3)		

Symmetry codes: (i) *x*, −*y*+1/2, *z*.
